# Computational support for a scaffolding mechanism of centriole assembly

**DOI:** 10.1038/srep27075

**Published:** 2016-06-08

**Authors:** Heinrich C. R. Klein, Paul Guichard, Virginie Hamel, Pierre Gönczy, Ulrich S. Schwarz

**Affiliations:** 1 Institute for Theoretical Physics and BioQuant, Heidelberg University, D-69120 Heidelberg, Germany; 2 Swiss Institute for Experimental Cancer Research (ISREC), School of Life Sciences, Swiss Federal Institute of Technology Lausanne (EPFL), CH-1015 Lausanne, Switzerland

## Abstract

Centrioles are essential for forming cilia, flagella and centrosomes. Successful centriole assembly requires proteins of the SAS-6 family, which can form oligomeric ring structures with ninefold symmetry *in vitro*. While important progress has been made in understanding SAS-6 protein biophysics, the mechanisms enabling ring formation *in vivo* remain elusive. Likewise, the mechanisms by which a nascent centriole forms near-orthogonal to an existing one are not known. Here, we investigate possible mechanisms of centriole assembly using coarse-grained Brownian dynamics computer simulations in combination with a rate equation approach. Our results suggest that without any external factors, strong stabilization associated with ring closure would be needed to enable efficient ring formation. Strikingly, our simulations reveal that a scaffold-assisted assembly mechanism can trigger robust ring formation owing to local cooperativity, and that this mechanism can also impart orthogonalilty to centriole assembly. Overall, our findings provide novel insights into the organizing principles governing the assembly of this important organelle.

Supramolecular protein complexes are central to many cellular processes^1^,^2^. Therefore understanding the mechanisms governing their coordinated assembly in a given cellular location and at the proper time is a fundamental pursuit in biology^3^. Centriole assembly exemplifies this general question^4^. The centriole is an evolutionarily conserved cylindrical macromolecular structure with a signature ninefold radial symmetry of microtubules. In most proliferating cells, a new centriole starts to assemble approximately at the G1/S transition, close to the proximal end of each of the two existing centrioles and with a near-orthogonal orientation^4^,^5^,^6^,^7^,^8^,^9^,^10^. Moreover, in specialized multiciliated epithelial cells, numerous centrioles can assemble orthogonal to the surface of a spherical structure called the deuterosome^11^,^12^,^13^,^14^. The mechanisms ensuring that centriole assembly occurs in a spatially and temporally restricted manner, as well as those imparting orthogonality to the newly emerging structure, remain elusive.

The characteristic ninefold radial symmetry of centrioles stems in most systems from the so-called cartwheel, which consists of several rings with a central hub ~23 nm in diameter, from which nine spokes point radially outwards^9^,^15^,^16^,^17^. SAS-6 proteins are key for cartwheel formation from algae to men^4^,^18^,^19^,^20^,^21^. They consist of a globular N-terminal (N-term) domain followed by an extended coiled-coil (CC) and an unstructured C-terminal part. Homodimerization of SAS-6 proteins is mediated by a relatively strong interaction between the CCs, with an equilibrium dissociation constant 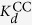 of ~1 *μ*M for the *Chlamydomonas reinhardtii* SAS-6 protein Bld12p (hereafter referred to as CrSAS-6)^22^ (Fig. 1a). Experiments in human cells revealed that human SAS-6 (HsSAS-6) exists predominantly in its homodimeric state in the cytoplasm^23^. SAS-6 proteins can undergo further oligomerization driven by an interaction between the N-term domains of SAS-6 homodimers^22^,^24^,^25^. Such further interaction is considerably weaker than that between the CCs, with an equilibrium dissociation constant 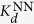 of ~60 *μM* for two individual N-term domains of CrSAS-6^22^,^24^ (Fig. 1a). Nine homodimers of SAS-6 proteins can assemble *in vitro* into ring-like structures that resemble the central hub of the cartwheel^17^,^22^,^24^,^25^,^26^, suggesting that proteins of the SAS-6 family act as a nucleus for the assembly process and thus dictate the signature ninefold symmetry of the entire centriole. The concentration of HsSAS-6 has been estimated to be in the order of 0.1 *μ*M in the cytoplasm and in the order of 5–10 *μ*M in the centrosomal region^23^ (Fig. 1b).

While aspects of the structure and the bimolecular interactions of SAS-6 proteins have been extensively investigated, the mechanisms enabling the assembly of complete rings at physiological concentrations remain elusive. The fact that CrSAS-6 expressed in *E. coli* can form rings *in vitro* seems to suggest that self-assembly properties provided by SAS-6 proteins can be sufficient^22^,^24^. For productive ring assembly in the cellular context, however, additional factors might be required, e.g. post-translational modifications of SAS-6 proteins or binding to additional components. One intriguing possibility in this context is that scaffolds may help direct the assembly of SAS-6 rings. For instance, such a role may conceivably be played by the Cartwheel Inner Densities (CID) of yet unknown molecular composition that have been observed within the central hub of the cartwheel in *Trichonympha sp.*, the sole organism for which a 3D architectural map of the cartwheel is available^17^. We set out to address the mechanisms underlying SAS-6 ring assembly by using coarse-grained Brownian dynamics (BD) simulations in combination with a rate equation approach. Our main results are that physiological concentrations of SAS-6 make ring formation very unlikely and that scaffold-assisted mechanisms indeed might be used in cells to ensure centriole assembly. Our computational analysis also suggests a simple geometrical mechanism to explain near-orthogonality of centriole assembly.

## Methods

### Coarse-grained model for SAS-6

In order to simulate the assembly of SAS-6 rings, we use recent advances in particle-based stochastic computer simulations^27^,^28^,^29^,^30^,^31^. Particle-based simulations combine molecular information with reasonable computing times and therefore fill the gap between time-consuming molecular dynamics (MD) simulations with atomistic details and reaction-diffusion models that are efficient but carry only little molecular information. To model spatially extended protein architectures, we represent proteins as anisotropic particles with reaction patches (*patchy particles*)^32^. Because cytoplasmic HsSAS-6 exists predominantly in its homodimeric state^23^ and because the CC interaction leading to homodimerization is ~ 60 times stronger than that between two N-term domains^22^, homodimers are considered as the smallest assembly unit.

**Figure 2:**
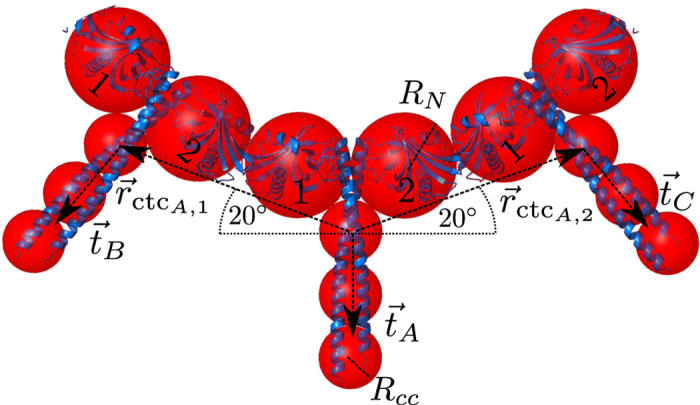
Superposition of the experimentally determined CrSAS-6-6HR ring structure with the coarse-grained model for three SAS-6-6HR homodimers, each consisting of five spheres. In the model the two globular N-term domains are described by a dumbbell consisting of two spheres of sizes *R*_N_ = 1.979 nm and the first six heptad repeats by a string of three spheres of size *R*_CC_ = 1.182 nm. In our coarse-grained model the ninefold planar ring structure is encoded by the center-to-center vectors 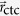 and the torsion vectors 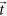, as indicated in the figure.

We first built a rigid patchy particle model for the homodimer starting from the CrSAS-6 Protein Data Bank (PDB) structures 3Q0X and 3Q0Y, which contain six heptad repeats of the CC. Hereafter the corresponding experimental and model structures are denoted as CrSAS-6-6HR and SAS-6-6HR, respectively. The two N-term domains of a homodimer are represented by a dumbbell consisting of two spheres and the first six heptad repeats of the CC by a string of three smaller spheres (Fig. 2). As oligomerization of homodimers is mediated by an interaction between localized binding sites situated in the N-term domains^22^,^24^, as a next step each SAS-6-6HR homodimer was equipped with two corresponding reaction patches (Fig. S1b). The reaction patches bind neighbors by bonds that implement a 40 degree angle between adjacent spokes. Center-to-center vectors and torsion vectors were defined such that planar rings can assemble, with spokes pointing radially outwards (Fig. 2). This procedure has been described before for generic fivefold rings in a computational study of patchy particle assembly^32^.

It has been found experimentally that CrSAS-6 can assemble not only into ninefold rings, but also sometimes into eight- or tenfold rings, indicating some degree of flexibility^26^. Using MD-simulations, it has been further shown that the observed distribution might result from the natural fluctuations in the binding angle between two homodimers. Here we focus on the statistics of ring assembly and therefore neglect the variability in binding angle and ring size, which *in vivo* is expected to be further diminished by the interplay with the microtubule array^26^.

### Spatially resolved Brownian dynamics simulations

To simulate SAS-6 ring assembly in a spatially resolved manner, an approach combining Brownian dynamics (BD) and localized stochastic reactivity was used^32^. Homodimers and their oligomeres are treated as rigid objects and propagated between reactions according to their anisotropic translational and rotational diffusive properties^33^,^34^,^35^, which are evaluated on-the-fly^36^,^37^. An association reaction is conceptualized as a two-step process: first, diffusive motion until an encounter and, second, transition from this encounter to a bound state^38^,^39^. The encounter state is defined by a range of relative configurations (see Eqs S11–S14) around the expected bound configurations. Two clusters forming an encounter can stochastically react with probability 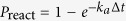, where *k*_*a*_ is the microscopic association rate and Δ*t* the time step (typically a nanosecond or less). If a bond is established, the two reacting clusters assume the relative position and orientation defined by the set of local rules encoded in the center-to-center and torsion vectors, provided no self-overlap in the bound configuration occurs. Otherwise the system is set back to its previous configuration. Overlaps with other clusters are resolved by additional propagation steps of the newly formed clusters during which a repulsive force acts on the centers of the spheres overlapping with other clusters or with non-periodic boundaries. To ensure reversible reaction dynamics, it is assumed that every existing bond can dissociate with probability 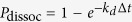, where *k*_*d*_ is the microscopic dissociation rate. During this step unconnected clusters are positioned relative to each other in such a way that detailed balance is satisfied^32^.

Binding of the last open bond in a ring plays a special role because, in contrast to the others, it does not result from a diffusional encounter and thus is expected to be faster than the other binding steps^32^. Thus a special rate 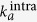 is attributed to the formation of the last bond. The free energy associated with this stabilization of the complete ring is related to the microscopic rate by


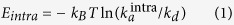


with *k*_*B*_ being the Boltzmann constant and *T* being temperature. Once the last bond has closed, every dimer in the ring is connected to two neighbors and two bonds need to open simultaneously for the ring to break apart. The dissociation constant *k*_*d*_ is assumed to be the same for all bonds.

### Scaffold-assisted assembly

We model scaffolds as rigid bodies of either cylindrical or spherical shape. They interact with SAS-6 proteins by a steep soft core potential starting at a radius *R*_*s*_ (see Fig. S1a). While the steepness of the potential ensures that we essentially model hard core repulsion, the soft part ensures that our algorithm deals correctly with detailed balance. The repulsive part is surrounded by an attractive layer of width Δ*R*_*s*_. The layer around the inner core exerts an attractive force on the assembling proteins:


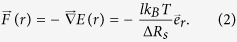


The strength of this interaction is defined in units of *k*_*B*_*T* and regulated by the dimensionless parameter *l*. The forces exerted on the cluster by the interaction with the scaffold result in an additional drift term in radial direction biasing the translational and rotational motion of the clusters. To simulate assembly assisted by a cylindrical scaffold, which extends periodically in the *z*-direction, at a desired concentration and without monomer starvation, the simulations are coupled to a particle reservoir by adapting a method based on grand-canonical Monte Carlo (GCMC) steps^40^. To avoid unphysical correlations, the GCMC steps are restricted to a region far away from the scaffold (the exchange region) and the time between them is short compared to that a SAS-6-6HR homodimer needs to diffuse through the exchange region^40^,^41^,^42^. Simulations with (semi-)spherical scaffolds are performed without coupling to a reservoir.

### Parameter choice and rate equation approach

Two different sets of parameters have been used (Table SI). In both cases, the microscopic reaction rates *k*_*a*_ and *k*_*d*_ are chosen to reflect the experimentally measured equilibrium dissociation constant for CrSAS-6^22^. Bulk assembly was simulated with parameter set 1, in which the patch parameters are specified so as to reproduce the diffusive reaction rate constant (speed of the assembly process) predicted by the TransComp-webserver^43^,^44^,^45^ for two N-term domains at physiological salt conditions (Fig. S3 and associated discussion). In order to be able to simulate complete ring assembly using parameter set 1, we used a rate equation approach that has been shown before to give very similar results for ring assembly as do the spatially resolved BD-simulations^32^. The diffusive reaction rate constants needed for the rate equation approach are calculated from short BD-simulations^32^,^46^,^47^. *k*_*a*_ is chosen sufficiently large so that the reaction process can be considered as diffusion-limited^48^. In contrast to bulk assembly, scaffold-assisted assembly cannot be treated with a rate equation approach and therefore in this case BD-simulations are used to describe the complete process. It thus required more computer time and therefore was simulated using parameter set 2. Here patch parameters allow for reactions in a larger range of relative configurations, resulting in faster assembly dynamics (Table SV). Accelerating the assembly dynamics in this way can be understood as a rescaling the time for bulk assembly (Fig. S4).

## Results

### Bulk assembly

**Figure 3:**
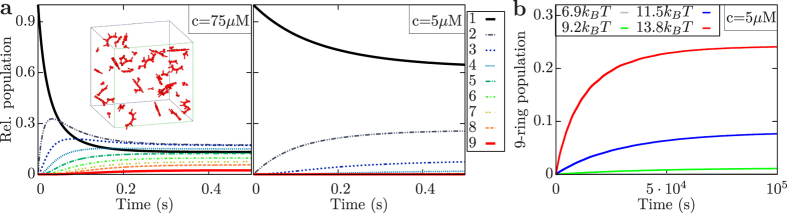
Bulk assembly simulated with the rate equation approach. (**a**) Diffusion-limited evolution of the relative cluster population without ring stabilization for *c* = 75 *μ*M (left plot) and *c* = 5 *μ*M (right plot). (**b**) Effect of different stabilization energies for ring closure on the fraction of complete rings for *c* = 5 *μ*M.

We set out to investigate the mechanisms enabling SAS-6 ring formation. At first we considered a situation without any stabilization from ring closure 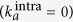, which becomes only relevant if complete rings can occur. Previous analytical ultra-centrifugation (AUC) experiments performed with bacterially expressed CrSAS-6-6HR at a concentration of *c* = 75 *μ*M suggest that at steady state, mainly two or three homodimers are present in higher order assemblies (Fig. S4 of reference^22^). We set out to test whether the model developed here reaches similar conclusions. Figure 3a (left) shows the evolution of the relative cluster population *p*_*i*_ in the model, which is the probability that a homodimer is part of an oligomer of size *i*, for a homodimer concentration of *c* = 75 *μ*M (see also supplementary movie 1). We find that the steady state is established on the sub-second time scale, with oligomeres of two and three homodimers being most frequent, in line with the AUC experiments.

Observing good qualitative agreement between the model and the experimental data, we turned to analyze the assembly reaction at physiological concentrations, which have been estimated to be in the order of *c* = 5–10 *μ*M at the centrosomes of human cells at the onset of centriole formation^23^. For simplicity, a concentration of *c* = 5 *μ*M will be considered hereafter. For this concentration of homodimers, we find that only small clusters form, with single homodimers being by far the most frequent state (Fig. 3a, right). We conclude that the formation of complete SAS-6 rings at the estimated physiological concentrations should not occur without additional stabilization mechanisms.

We next investigated which free energies associated with ring closure would be required to observe a significant fraction of complete rings at a concentration of *c* = 5 *μ*M. Figure 3b reports the evolution of the relative population of complete rings for different free energies (see Eq. 1). We first note that the time scale for assembly of complete rings is much longer than for small oligomers. We then note that a stabilization free energy of *E* ≈ −9.2 *k*_*B*_*T* 
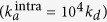
 leads only to a slight increase in the fraction of complete rings (to ~ 1 percent). For a more substantial increase (to ~ 25 percent), a stabilization energy of *E* ≈ −13.8 *k*_*B*_*T* would be needed. Such a gain in free energy is larger than the values for the association free energies per contact determined for various viral capsids^49^ and even with this strong stabilization the time scale for assembly would be hours. We also note that the free energies required to obtain a substantial proportion of closed rings at *c* = 5 *μ*M would result in a very high proportion of complete rings at *c* = 75 *μ*M (Fig. S5), which has not been observed experimentally^22^. Overall, we conclude that SAS-6 ring formation at physiological concentrations seems unlikely to be explained solely by the stabilization properties of complete rings.

### Assembly assisted by a cylindrical scaffold

**Figure 4:**
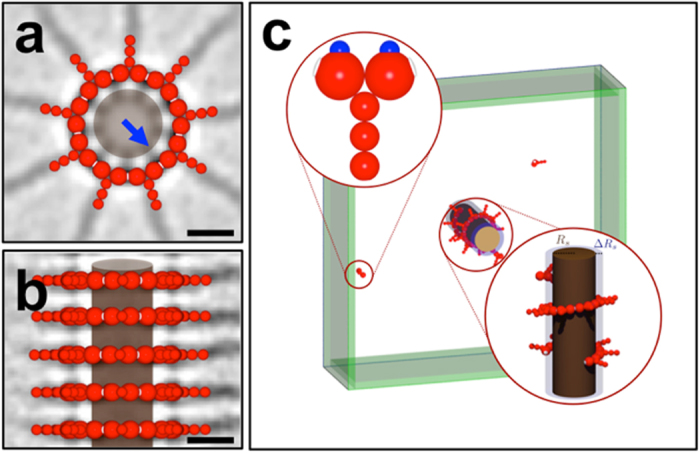
(**a**,**b**) Superposition of the cryo-EM image of the central hub of the cartwheel in *Trichonympha*^17^ with the coarse-grained CrSAS-6-6HR model (red). In the cross section (**a**), one sees the Cartwheel Inner Densities (CID) that connect to all SAS-6 heads (blue arrow). The brown cylinder shown here and in the side view (**b**) is our model for an inner scaffold. Scale bars 10 nm. (**c**) Simulation snapshot of the cylindrical scaffold (repulsive inner core *R*_*s*_ = 9 nm surrounded by an attractive layer of width Δ*R*_*s*_ = 3 nm) which is embedded in a simulation volume of size 200 × 200 × 60 nm^3^. The two charges located on top of the N-term domains (blue) experience a constant force within the attractive layer. The simulation volume is coupled to a particle reservoir by GCMC steps restricted to the region shown in green (width 5 nm).

Whereas the above conclusions result from considering unmodified SAS-6 proteins on their own, other factors are likely to contribute to ring formation *in vivo*. As an extension of our computational framework, we set out to investigate the possibility that a scaffolded assembly mechanism may be important, inspired by the presence of the CID within the central hub of the cartwheel in *Trichonympha*^17^. Because the CID abuts the interface between two homodimers of SAS-6 proteins (Fig. 4a, blue arrow)^17^, we reasoned that this may serve to stabilize ring formation. Therefore, we next analyzed ring assembly assisted by an interaction of the N-term domains with an inner scaffold. Figure 4a,b shows the CID inside the cylindrical cartwheel as observed in *Trichonympha*, together with the coarse-grained model of SAS-6-6HR. To represent the tubular geometry of the CID, a cylindrical scaffold is used (*R*_*s*_ = 9 nm, Δ*R*_*s*_ = 3 nm), which is centered in the x-y plane in a simulation volume of size *V*_sim_ = 200 × 200 × 60 nm^3^ and extends periodically in the z-direction. The attractive interaction between scaffold and SAS-6-6HR homodimers is modeled by two charges located on top of the N-term domains that correspond to where the CID is known to abut the N-term domains (Fig. 4a, blue arrow). In addition, for this setup the simulation volume is coupled to a particle reservoir with concentration *c*_*r*_ by GCMC steps which are restricted to the region shown in green in Fig. 4c (width of 5 nm). In order to focus solely on the potential contribution of a scaffold, in the following no stabilization free energies are being considered 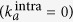.

**Figure 5:**
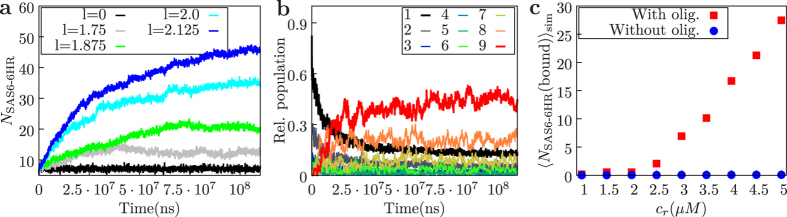
Assembly around a cylindrical scaffold simulated with Brownian dynamics. (**a**) Evolution of the number of homodimers in the simulation volume (irrespective of their oligomerization state) for different interaction strengths *l* and a reservoir concentration of *c*_*r*_ = 5 *μ*M. (**b**) Relative cluster population for *l* = 2 and *c*_*r*_ = 5 *μ*M. (**c**) Comparison of the concentration dependence of the number of SAS-6-6HR homodimers bound to the cylinder in steady state with oligomerization (red) and without oligomerization(blue) for *l* = 2.

We first examined the behavior of such a scaffold-assisted system for different interaction strengths *l* between scaffold and SAS-6-6HR clusters at *c*_*r*_ = 5 *μ*M (see Eq. 2). As shown in Fig. 5a, we find that augmenting interaction strengths results in a strong increase in the number of homodimers present in the simulation volume, irrespective of their oligomerization state. This indicates that a threshold in interaction strength is needed for efficient capture of SAS-6-6HR homodimers by the cylinder. Once a steady state is reached, the number of newly captured SAS-6-6HR homodimers is balanced by those breaking free from the cylinder. Importantly, the cylindrical scaffold also has a strong effect on the observed oligomerization state (Fig. 5b). Thus, for an attractive interaction of *l* = 2, it is most probable that a SAS-6-6HR homodimer is part of a complete ring. Overall, we conclude that a cylindrical scaffold can efficiently assist the formation of complete rings by enhancing encounter configurations for clusters bound to the cylinder.

As indicated by Fig. 5a, a threshold of interaction strength needs to be surpassed before a significant number of homodimers is captured by the cylindrical scaffold. Importantly, we also find that the capturing capability of the cylinder depends on the ability of SAS-6-6HR homodimers to oligomerize (Fig. 5c). This can be explained by the fact that formation of larger clusters has a strong influence on the detachment probability from the cylinder: the net force driving oligomers towards the cylinder increases with their size, resulting in a lower detachment rate from the cylinder due to cooperativity. Interestingly, the dependence of the detachment rate on cluster size results in a positive feedback: the larger the cluster the longer it stays bound to the cylinder, which in turn increases chances for further oligomerization. This local cooperativity enables a steep response of the system to relatively small changes in the surrounding concentration (Fig. 5c and Fig. S7), which might be critical for robust centriole formation.

### Assembly assisted by spherical and semi-spherical scaffolds

The CID has been observed only in *Trichonympha* thus far, but our results raise the possibility that an analogous scaffolding mechanism might also play a crucial role for the assembly of canonical centrioles. As new centrioles grow from the outer surface from the existing centrioles, one might expect that the corresponding scaffolds are more compact in this case than the elongated CID in *Trichonympha*. For example, it has been found experimentally that a Cep152/Cep63/Cep57-containing torus surrounds the proximal end of existing centrioles, onto which a focus of PLK4 is found, from which new centrioles emerge in human cells^4^,^9^,^10^,^50^. Likewise, a torus containing the Cep63 paralogue Deup1 present in deuterosomes seeds the formation of centrioles in multiciliated cells^13^.

**Figure 6:**
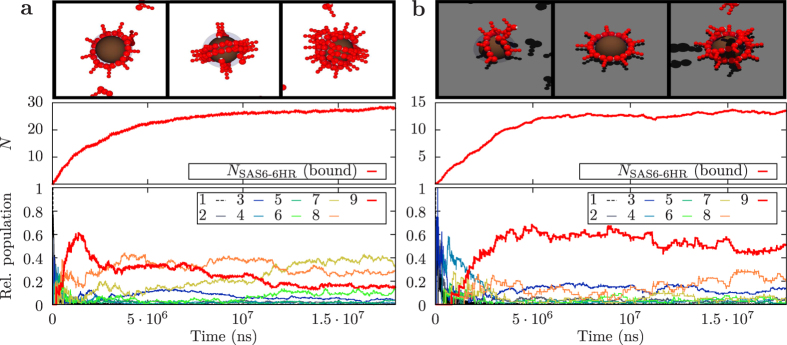
Assembly around a compact scaffold for an interaction strength of *l* = 4 and a concentration of *c* = 5 *μ*M simulated with Brownian dynamics. Simulation snapshots, numbers of bound SAS-6-6HR homodimers and frequencies of different cluster sizes for a freely accessible spherical scaffold (**a**) and for a semi-spherical scaffold on a planar surface (**b**).

We simulated spherical and semi-spherical scaffolds representing the locations within the cell at the proximal end of each existing centriole from where the new centriole emerges. Like for the cylindrical scaffold, the spherical scaffold is defined by an inner radius of *R*_*s*_ = 9 nm surrounded by an attractive layer of width Δ*R*_*s*_ = 3 nm. Simulations are started with 150 randomly placed homodimers inside a simulation volume of size 368 nm^3^, corresponding again to a concentration of *c* ≈ 5 *μ*M. An interaction strength of *l* = 4 was chosen in this case, which is higher than the one used for the cylindrical scaffold, because the attractive surface of the sphere is smaller. In a first setup, the spherical scaffold is placed in the center of the periodic simulation volume (Fig. 6a, top; see also supplementary movie 2). In a second configuration, the semi-spherical scaffold is embedded in an otherwise planar at *z* = 0 (Fig. 6b, top; see also supplementary movie 3).

We compared the ability of the scaffold to assist the assembly process in both setups by monitoring the evolution of the number of SAS-6-6HR homodimers attracted to the surface (Fig. 6). We find that this number is higher in the case of the freely accessible scaffold. This is not surprising given that the accessible surface is twice as large in this case. Unexpectedly, however, we find that for the freely accessible spherical scaffold, the fraction of rings first increases and then decreases again (Fig. 6a). At later times, many randomly oriented large clusters are present on the scaffold. In marked contrast, in the case of the semi-spherical scaffold, the symmetry break prevents the formation of multiple large clusters and complete rings are assembled very robustly (Fig. 6b). Remarkably, we found that such rings are oriented parallel to the surface, potentially offering a mechanism through which a small embedded globular seed that leaves a semi-sphere accessible to SAS-6 proteins could direct assembly of the new centriole orthogonal to the existing one or to the deuterosome.

## Conclusions

The mechanisms governing centriole formation remain incompletely understood, despite important progress in recent years regarding the identification and structural characterization of participating proteins. Proteins of the SAS-6 family play a particularly critical role in centriole formation as they can assemble into ninefold symmetrical ring structures and are thought to be the main constituents of the cartwheel, which is pivotal for determining the striking symmetry of the whole organelle^9^,^15^,^16^,^17^,^21^,^22^,^51^,^52^.

In this work we have investigated possible assembly mechanisms for the formation of complete rings at physiologically relevant concentrations of SAS-6 proteins. Our results indicate that unassisted ring assembly at concentrations estimated at centrosomes at the onset of centriole formation in human cells^23^ is only possible if assuming ring closure to be associated with a high gain in free energy. Moreover, the assembly time for complete rings would be very large. Post-translational modifications of SAS-6 proteins as well as interactions with associated proteins such as Bld10p in *Chlamydomonas* or STIL in human cells may effectively contribute towards such a large gain in free energy *in vivo*. Our analysis suggests another mechanism that might help explain how SAS-6 rings can form *in vivo*: scaffold-assisted assembly. Indeed, our simulations show that this mechanism leads to strong local cooperativity, which renders assembly very sensitive towards small changes in the surrounding protein concentration. Thus, scaffold-assisted assembly of SAS-6 rings could account for the tight regulation of centriole formation onset that occurs approximately at the G1/S transition of the cell cycle. Remarkably, our simulations reveal that placing a semi-spherical scaffold on a planar surface leads to very robust ring formation, and ensure that the cartwheel would emerge orthogonal to this surface. It has been clear since the advent of electron microscopy that new centrioles emerge near-orthogonal to existing ones during the canonical duplication cycle, as well as to the deuterosome in multiciliated cells^5^,^6^,^7^,^8^,^10^, but the root of this geometrical feature has remained mysterious. We propose that a scaffold-assisted assembly mechanism may be at the origin of this remarkable geometry of centriole formation.

Interestingly, centrioles can also assemble de novo in some cases, for instance following microsurgical removal of resident centrioles from human cells, although slower than normally^53^. Such de novo formation occurs around an electron dense amorphous cloud^53^ and we speculate that this may also serve as a scaffold to direct the assembly of HsSAS-6 rings. While here we have investigated the effect of a central scaffold assisting assembly by an interaction with the N-terminal domain, another mechanism has been proposed in which the existing centriole acts as blueprint for an interaction with the coiled-coil domain and/or the C-terminal domain^54^. However, no structural information exists at present on the C-terminal domain of SAS-6 proteins, which makes it difficult to computationally model possible mechanisms.

The coarse-grained SAS-6-6HR model used here harbors a shorter coiled-coil extension (~7 nm) than native SAS-6 proteins (~50 nm). However, extending the coiled-coil in our model by the addition of further spheres led to only minor changes in our results (Fig. S6). This was expected from the fact that longer coiled-coil should mainly change the kinetic (diffusive) properties and not the affinities. Thus the results obtained with our model for SAS-6-6HR ought to be applicable also to SAS-6 homodimers with a native coiled-coil length provided no additional interactions between the coiled-coil domains occur.

We note that our simulation approach is very general and in the future might be extended to simulate other aspects of centriole assembly, for instance the addition of more distal segments of the organelle or that of centriolar microtubules that co-assembles with the SAS-6 ring^26^. We also note that without the combination of Brownian dynamics and rate equations used here, we could never simulate the large assembly times for complete rings.

Interestingly, scaffold-assisted assembly is a common process in biological systems, with maybe the most prominent example being viruses in which the viral protein capsid assembles around the genomic material or along the plasma membrane^55^,^56^. Like for viral assembly, scaffolds for centriole assembly might be very effective because they lead to strong cooperative effects and robust assembly. At the same time, they need not to be particularly organized and could result from a simple physical condensation process, as examplified here on the surface of the existing centriole.

## Additional Information

**How to cite this article**: Klein, H. C. R. *et al.* Computational support for a scaffolding mechanism of centriole assembly. *Sci. Rep.* **6**, 27075; doi: 10.1038/srep27075 (2016).

## Supplementary Material

Supplementary Information

Supplementary Movie 1

Supplementary Movie 2

Supplementary Movie 3

## Figures and Tables

**Figure 1:**
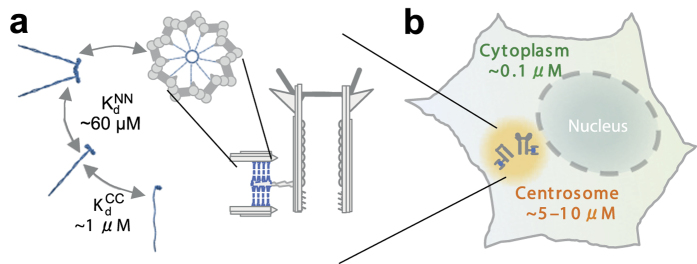
(**a**) Side view (right) and cross section (top) of a new centriole assembling near-orthogonal to an existing one. Both the cartwheel (in blue) and the microtubules (in gray) have a ninefold symmetry that is thought to be imparted in large part by the self-assembly of SAS-6 proteins (left). The binding affinities for homodimerization through the coiled-coil domains 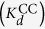 and for oligomerization through the N-term domains 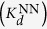 have been measured *in vitro* to have the values shown. (**b**) Experimentally estimated concentrations of HsSAS-6 in the cytoplasm and at the centrosome of human cells.
