# Supplementary material for: Computational support for a scaffolding mechanism of centriole assembly
Source: Sci Rep. 2016 Jun 8;6:27075. doi: 10.1038/srep27075 (PMC4897622; doi:10.1038/srep27075)
Supplement: Supplementary Information [file srep27075-s1.pdf]

Supplementary Information
Computational support for a scaffolding mechanism of centriole assembly

Heinrich C. R. Klein, Paul Guichard, Virginie Hamel, Pierre Gönczy and Ulrich S. Schwarz

I. BROWNIAN DYNAMICS SIMULATIONS

To achieve reasonable simulation times, we use particle-based stochastic computer simulations. In contrast to molecular dynamics simulations, we do not explicitly include atomistic details or molecular flexibility. Protein assemblies continuously react with each other and form larger or smaller clusters. A given cluster is treated as a rigid body that translates and rotates in space under the influence of thermal forces. The configuration of such a cluster can be described by a six-dimensional vector $\mathbf{X}$ containing the information about the position and orientation of the cluster. Below we use boldface and vector notations for six-dimensional configuration vectors and three-dimensional position vectors, respectively. The evolution of the configuration of a cluster is governed by the six-dimensional, overdamped Langevin equation, which in the force-free case reads [1–3]:

$$\partial_t \mathbf{X}_t = \mathbf{g}_t \tag{S1}$$
$$\text{with: } \langle \mathbf{g}_t \rangle = 0,\ \langle \mathbf{g}_t \mathbf{g}_{t'} \rangle = 2k_B T \mathbb{M}(\mathbf{X}_t)\delta(t-t').$$

Eq. S1 describes the stochastic change in the position and orientation of the cluster. Here $\mathbf{g}_t$ represents Gaussian white noise with the magnitude of the noise being defined by the 6×6-dimensional self-mobility matrix of the rigid cluster $\mathbb{M}$, which is related to the self-diffusion matrix of the cluster $\mathbb{D}$ by a Boltzmann factor. As clusters continuously grow and shrink due to association and dissociation events, also the self-diffusive properties of the clusters change during a simulation. Thus, the self-diffusion matrix $\mathbb{D}$ of a cluster is evaluated on-the-fly upon its generation [3–5]. The dependence of the self-mobility matrix on the cluster configuration in Eq. S1 reflects the fact that the self-mobility matrix is only constant in a frame moving with the cluster. In a frame which is fixed in space (called the laboratory frame below), it depends on the orientation of the cluster. Evaluating the change in configuration with respect to a cluster-fixed frame, the self-mobility matrix of the cluster becomes independent of its configuration and the noise contribution is purely additive. In our approach clusters are propagated with a finite timestep increment Δt. Hence, we use a time-discretized version of Eq. S1 and evaluate the change of the configuration of a cluster during the timestep $\Delta t = t - t_0$ with respect to a cluster-fixed frame at time t_0 which is denoted by the prime symbol [1–3, 6]:

$$\Delta \mathbf{X}' = \mathbf{g}(\Delta t) + O(\Delta t^2) \tag{S2}$$
$$\text{with } \langle \mathbf{g}(\Delta t) \rangle = 0,\ \langle \mathbf{g}(\Delta t)\mathbf{g}(\Delta t) \rangle = 2k_B T \mathbb{M}\Delta t.$$

As Eq. S2 describes the change in configuration of a cluster with respect to a cluster-fixed frame at time t_0, we have to transform the configuration update of the cluster in the laboratory coordinate system. This is indicated by the generalized rotation matrix $\mathbb{R}_6$, which acts on both, the position and orientation:

$$\mathbf{X}(t+\Delta t) = \mathbf{X}(t) + \mathbb{R}_6 \Delta \mathbf{X}'. \tag{S3}$$

The details of our update scheme for the orientation coordinates has been described elsewhere [1]. Note that by considering only the self-diffusion properties of a cluster we neglect hydrodynamic interactions.

To investigate the effect of a localized scaffold assisting the formation of complete SAS-6 rings, we model the interaction between the scaffold structure and clusters by repulsive and attractive forces acting between the scaffolding structure and a particular region of the SAS-6 homodimer. In Fig. S1a a schematic drawing of the cylindrical and spherical scaffolds used in the simulations is shown. Each scaffold is defined by an inner core with radius R_s and surrounded by an attractive layer of width ΔR_s. The attractive force between scaffold and the SAS-6-6HR homodimers is mediated by two point-like charges located on the top of the N-term domains (see Fig. S1a). A charge which is inside the attractive layer ΔR_s experiences a force according to Eq. 2. This force results in an additional drift term biasing the translational and rotational motion of the clusters, and the configuration update for a cluster according to Eq. S2 now reads [1–3]:

$$\Delta \mathbf{X}' = \mathbb{M}\mathbf{F}'(t)\Delta t + \mathbf{g}(\Delta t) + O(\Delta t^2). \tag{S4}$$

In Eq. S4 $\mathbf{F}' = (\vec{F}', \vec{\tau}')$ is a generalized force vector which contains the three-dimensional force and the torque acting on the cluster. As the force $\vec{F}_i$ acting on a particular "charge" i, located at position $\vec{r}'_{c,i}$ relative to the cluster, is evaluated in the laboratory-fixed frame, the generalized force $\mathbf{F}'$ acting on the cluster in the cluster-fixed frame is calculated according to:

$$\vec{F}' = \sum_i \mathbb{R}_3^T \vec{F}_i\ ,\ \vec{\tau}' = \sum_i \vec{r}'_{c,i} \times \vec{F}'\ ,\ \mathbf{F}' = (\vec{F}', \vec{\tau}'). \tag{S5}$$

Here we sum over all charges of a cluster and $\mathbb{R}_3^T$ is the three-dimensional rotation matrix converting between the laboratory-fixed and cluster-fixed frame.

Similarly to the force acting on the charges, a repulsive Lennard-Jones type of force acts on a steric sphere overlapping with the inner region of the scaffold defined by the radius R_s (see Fig. 1a). The repulsive force acting

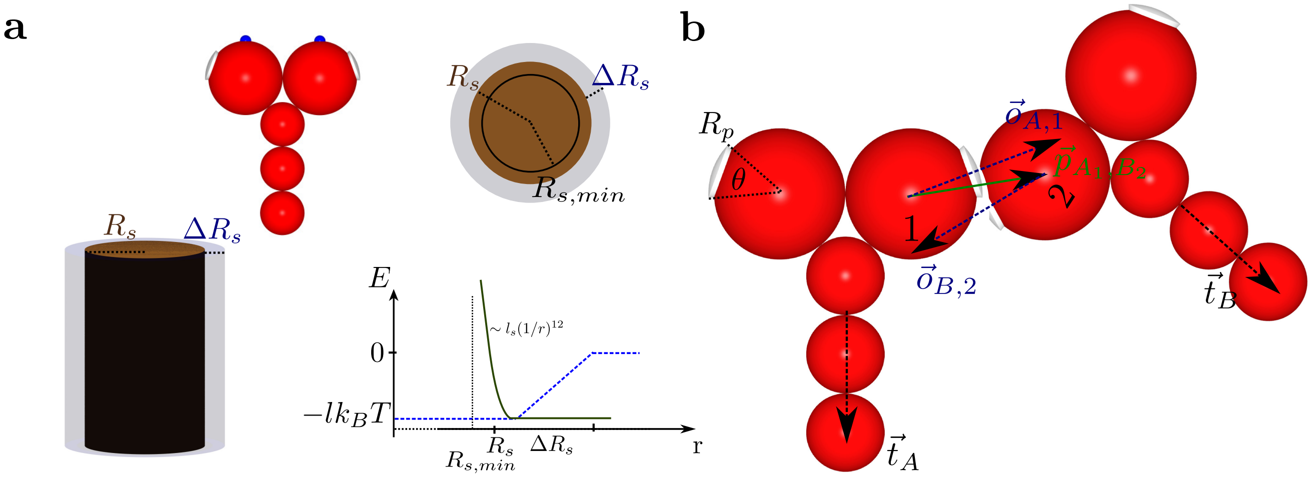

Figure S 1. (a) Sketch of cylindrical and spherical scaffold with inner radius R_s surrounded by attractive layer of width ΔR_s together with the SAS-6-6HR model with the charges attracted to the scaffold being shown in blue. In the bottom row a visualization of the potential with its repulsive and attractive parts is shown. (b) Illustration of an encounter complex between two homodimers A and B. The encounter is mediated by patch 1 of homodimer A and patch 2 of homodimer B. Patches are defined by a radius R_p and an angle θ. The orientation vectors $\vec{o}$ reflect the desired nine-fold symmetry and the torsion vectors ensure that the CC domains are radially pointing outwards.

on a steric sphere of radius R_{steric} is given by:

$$\vec{F}/(k_BT) = l_s/(R_s - R_{s,min}) \left(\frac{R_s - R_{s,min}}{R - R_{\text{steric}} - R_{s,min}}\right)^{13} \vec{e}_r \quad \text{(S6)}$$

In Eq. S6 l_s is a dimensionless parameter scaling the force, $\vec{e}_r$ is a normal vector pointing toward the center of the scaffold and $R_{s,min}$ marks an inner radius at which the potential becomes infinite. Thus, $R_{s,min}$ regulates the steepness of the soft-core repulsion. For the simulations performed here we used $l_s = 0.1$ and $R_{s,min} = 6$nm. These parameters ensure that neither large displacements during a simulation step due to very high repulsive forcing nor large intrusions of clusters inside the scaffold are observed. Similarly to the forces acting on the "charges" (Eq. S5), the resulting force and torque acting on the whole cluster due to this soft-core repulsion during a motion step are calculated by summing up all forces and torques acting on individual steric spheres of the cluster and rotating them into the cluster-fixed frame.

II. REACTION RATES

Our simulation approach is conceptually based on the encounter complex scheme in which a reaction is decomposed into a two-step process: the diffusive motion of the reactants A and B until they reach an encounter state $A \cdot B$ and the binding step from the encounter state into the bound complex C (e.g. [7–14]). Considering the encounter complex as the "watershed" between diffusion and reaction, a bimolecular reaction can be split according to the following reaction scheme:

$$A + B \underset{k_-}{\overset{k_+}{\rightleftharpoons}} C \quad \text{(S7)}$$

$$A + B \underset{k_{D,b}}{\overset{k_D}{\rightleftharpoons}} A \cdot B \underset{k_d}{\overset{k_a}{\rightleftharpoons}} C. \quad \text{(S8)}$$

While in Eq. S7 k_+ and k_- represent the overall kinetic rate constants of the reaction, in Eq. S8 the binding and unbinding process is split into a diffusive and reactive part. The diffusive part is described by the rate constants k_D and $k_{D,b}$ while the reactive part is described by the reaction rates k_a and k_d, respectively.

Macroscopically, the reaction scheme in Eq. S8 can be understood as a system of ordinary differential equations describing the changes in the concentrations c_A,c_B,$c_{A \cdot B}$ and c_C [8]. In this case the encounter complex $A \cdot B$ represents a single, intermediate state between the bound complex C and the free particles A and B. The encounter complex can either decay into two separated particles A and B with the first order rate constant $k_{D,b}$, or it can react to the bound complex with the first order rate constant k_a. Similarly, an encounter is formed by the first order decay of the complex C with a rate k_d or diffusively from the separated A and B particles, which is described with the second order rate constant k_D. For a 3-dimensional system k_D has physical units of m^{3}s^{-1} when considering particle concentrations, or M^{-1}s^{-1} when considering molar concentrations. Assuming the concentration of the encounter complex to be in a steady state, the following relations between the overall reaction rate constants used in Eq. S7 and reaction rate constants in

the encounter picture (Eq S8) can be derived [8]:

$$k_+ = \frac{k_D k_a}{k_a + k_{D,b}} \ , \ k_- = \frac{k_{D,b} k_d}{k_a + k_{D,b}} \tag{S9}$$

$$\Rightarrow K_{\text{eq}} = \frac{k_+}{k_-} = \frac{k_D}{k_{D,b}} \frac{k_a}{k_d}. \tag{S10}$$

From Eq. S9 we can distinguish between the extreme cases of diffusion-limited and reaction-limited reactions [8]. The diffusion-limited case is realized when $k_a \gg k_{D,b}$. In this case the forward reaction is purely determined by diffusion $k_+ \approx k_D$ while the reverse reaction rate becomes $k_- \approx k_{D,b} k_d / k_a$. On the contrary, the reaction is considered to be reaction-limited when $k_{D,b} \gg k_a$. In this case the rate constant for the forward reaction becomes $k_+ \approx k_D k_a / k_{D,b}$ while the overall rate of the reverse reaction equals the microscopic dissociation rate $k_- \approx k_d$. Why this case is referred to as reaction-limited becomes apparent when reconsidering the model introduced by Collins and Kimball [7]. In this case finite reactivity between two spherical particles in encounter is introduced by a radiation boundary condition in which the rate constant κ (termed k in reference [7]) relates the concentration at contact to the reactive flux. Comparing the escape probabilities in the kinetic and in the Fokker-Planck picture, Shoup and Szabo [15] suggested the following relation between κ and the reaction rates used in Eq. S8: $\kappa = k_D k_a / k_{D,b}$. Given this relation it becomes apparent that in the reaction-limited case the forward rate constant is determined solely by the reaction from the encounter to the final state $k_+ \approx \kappa$.

The basic relations between the microscopic reaction rates k_a and k_d, the diffusive reaction rate constants and the macroscopic equilibrium association constant have been discussed elsewhere [16]. Based on these considerations an important quantity relating the ratio of the microscopic reaction rates to the macroscopic equilibrium association constant is the encounter volume, which is the region in the two-particle configuration space within which reactions between clusters are possible. Based on the patch definition (patch radius R^p, orientation vector $\vec{o}$) and the torsion vector $\vec{t}$ used to specify the relative orientations in the bound state (see Fig. 1b) the following set of constraints is used to define an encounter mediated by the patches i and j of the proteins A and B (which can belong to a larger cluster):

$$|\vec{p}_{A_i,B_j}| \leq 2R^p \tag{S11}$$

$$\text{acos}\left(\frac{\vec{p}_{A_i,B_j} \cdot \vec{o}_{A_i}}{|\vec{p}_{A_i,B_j}||\vec{o}_{A_i}|}\right) \leq \theta \tag{S12}$$

$$\text{acos}\left(\frac{-\vec{p}_{A_i,B_j} \cdot \vec{o}_{B_j}}{|\vec{p}_{A_i,B_j}||\vec{o}_{B_j}|}\right) \leq \theta \tag{S13}$$

$$\text{acos}\left(\frac{(\vec{p}_{A_i,B_j} \times \vec{t}_A) \cdot (\vec{p}_{A_i,B_j} \times \vec{t}_B)}{|(\vec{p}_{A_i,B_j} \times \vec{t}_A)||(\vec{p}_{A_i,B_j} \times \vec{t}_B)|}\right) \leq \chi. \tag{S14}$$

Eq. S11 describes a relative proximity criterion for the N-term domains involved in the binding process. Eq. S12 and Eq. S13 restrict the relative orientation of the two binding clusters to a certain range around the orientation defined by the local rules. Furthermore, the torsion constraint defined in Eq. S14 accounts for the non-globular nature of the SAS-6-6HR homodimers and prevents large-scale reorientation of the CC during the binding step by requiring sufficient alignment of the CCs with the planar ring geometry. Based on the constraints given in Eq. S11-Eq. S14 the size of the encounter volume for one pair of patches can be calculated according to [16]:

$$V^{\text{SAS-6}^\star}_{A_i,B_j} = V^{\text{rad}}_{A_i,B_j} V^{\text{ori}}_{A_i,B_j} V^{\text{tor}}_{A_i,B_j} \tag{S15}$$

$$V^{\text{rad}}_{A_i,B_j} = \frac{4\pi}{3}((2R^p)^3 - (2R_{\text{N}})^3) \ ,$$

$$V^{\text{ori}}_{A_i,B_j} = \frac{1}{4}(1 - \cos(\theta))^2 \ , \ V^{\text{tor}}_{A_i,B_j} = \frac{\chi}{\pi}.$$

R_N is the radius of the spheres for the N-terms. Note that in comparison to reference [16] the additional term χ/π reflects the additional constraint given in Eq. S14 and corresponds to the fraction of configurations for which the alignment of the projection of the torsion vectors is smaller than χ.

For a fixed set of patch parameters (R^p, θ, χ) the equilibrium association constant of a reaction K_{eq} is related to the ratio of the microscopic reaction rates k_a and k_d by the size of the encounter volume. Owing to the fact that each SAS-6 homodimer has two N-term domains and that the homodimer is symmetric under rotation of 180° around the CC, four different patch combinations exist which can lead to bond formation between two homodimers. Hence the total encounter volume for the reaction between two homodimers is given by:

$$V^{\text{SAS-6}^\star}_{1,1} = 4V^{\text{SAS-6}^\star}_{A_i,B_j}. \tag{S16}$$

Similarly, taking the four different possible patch combinations for a reaction into account, the equilibrium dissociation constant for two homodimers becomes $K^{\text{SAS-6}}_{\text{d}} = (K^{\text{SAS-6}}_{\text{eq}})^{-1} = K^{\text{NN}}_{\text{d}}/4 \approx 15\mu\text{M}$. Finally we have to account for the different counting of particles in macroscopic theories and simulations. Our rate equation approach takes into account that macroscopic reaction rate theories consider indistinguishable particles while in our simulations all particles are distinguishable. As a consequence the macroscopic reaction rate constant for the reaction between identical particles is reduced by a factor of 1/2 compared to the reaction rate constant used in our simulations [16–18]. Thus, as a consequence the macroscopically observed equilibrium association constant for the dimerization of identical particles is only half as large as the equilibrium association rate constant used in our simulations. To take this into account, in the following we always set:

$$k_d = \frac{V^{\text{SAS-6}^\star}_{1,1} k_a}{2K^{\text{SAS-6}}_{\text{eq}}}. \tag{S17}$$

This choice of k_d results in the macroscopically expected equilibrium constant for the dimerization. However, for

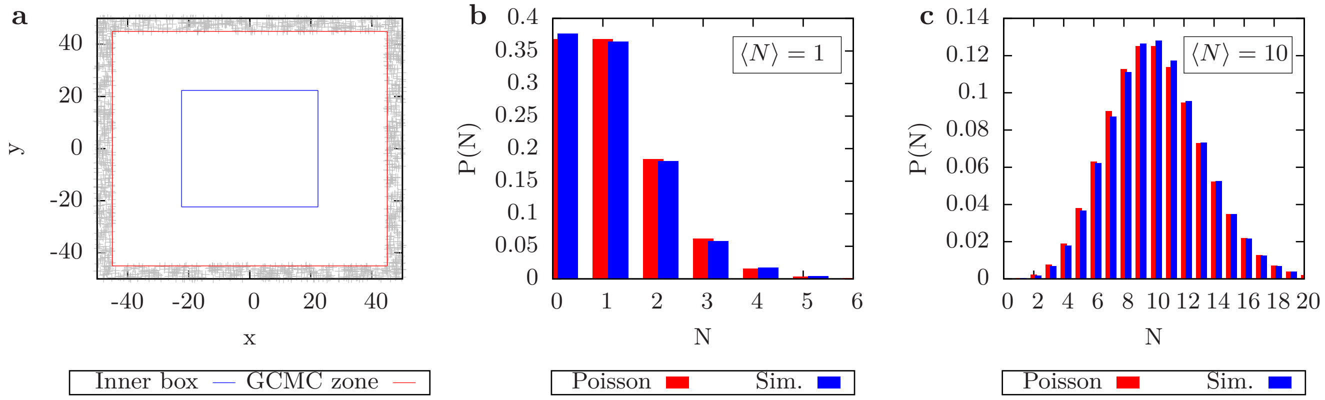

Figure S 2. (a) 2D projection of a simulation volume of size $(100\text{nm})^3$ used to illustrate the coupling mechanism. GCMC moves are restricted to a distance less than 5nm from the x- and y-boundary (shown by the red line), defining the GCMC subvolume V_{GCMC}. Gray marks depict the center of newly inserted proteins. The blue box marks an inner region. (b) and (c) show the comparison of the expected distribution (red) and the observed distribution (blue) of particles in the inner region of the setup shown in (a) for a reservoir particle concentration of $n_r = 5 \times 10^{-6}\text{nm}^{-3}$ and $n_r = 5 \times 10^{-5}\text{nm}^{-3}$, respectively.

non-identical ring fragments this choice might overestimate the binding fraction.

Knowledge of the size of the encounter volume and the experimentally measured equilibrium constant for N-term dimerization allows us to specify the ratio of the microscopic reaction rates so that the resulting equilibrium distribution of oligomers for a given concentration can be predicted based on the previously introduced rate equation approach [16]. However, in the diffusion-limited regime the time scale during which oligomerization takes place depends on the size (and shape) of the encounter volume as well as on the cluster geometries. Thus in order to determine the macroscopic rate constants k_+ and k_- defined in Eq. S9, the rate constant of the diffusive formation of the encounter k_D needs to be evaluated. This rate constant depends on the shape and diffusive properties of the clusters involved in the reaction and the size and shape of the encounter between them. Numerically, k_D can be calculated for two particular clusters based on a simulation algorithm proposed by Zhou [19]. In this algorithm the diffusive rate constants k_D and $k_{D,b}$ are evaluated from the survival probability $S(t)$ of two particles starting in a random encounter configuration by [13, 19, 20]:

$$k_D = \lim_{t\to\infty} \kappa \frac{S(t)}{1 - S(t)} \ , \ k_{D,b} = k_D / V^\star . \tag{S18}$$

In Eq. S18 $\kappa = V^\star k_a$.

III. COUPLING TO A PARTICLE RESERVOIR

Periodic boundaries are commonly used in particle-based simulations. However, in some cases one is interested to study a specific region embedded in a larger system. In this case simple periodic boundary conditions do not apply and the computational costs often prohibit simulations of a very large system with explicit particle resolution. This situation is given when investigating the effect of a cylindrical scaffold. While the scaffold is localized in the x-y plane, its height spans the whole z-extension of the simulation volume, and depending on the strength of the interaction between scaffold and SAS-6 homodimers, many homodimers can accumulate on the cylindrical surface. Thus, with simple periodic boundaries a very large extension of the simulation volume in x- and y- direction would be required to accommodate enough homodimers so that the effect of the scaffold at μM concentrations could be studied without starvation of the simulation volume. Studying such a large system is prohibited by the large computational costs of the simulations. Instead we couple our simulation volume to a homodimer reservoir. To couple BD simulation to a reservoir, hybrid schemes have been proposed in which the region explicitly described by BD simulations is coupled to a region described by density based methods [21–23]. While such an approach provides a mathematically solid framework to couple BD simulation with a continuous PDE-type description for point-like particles [23], a rigorous generalization to proteins with non-spherical shape is cumbersome. Thus, instead of using this coupling, we rely on another approach in which the simulation volume is coupled to a particle repository using grand canonical Monte Carlo (GCMC) steps, which are limited to a region far away from the region of interest [24–26]. This method can be generalized to extended proteins and clusters in the limit of dilute concentrations.

The basic idea of GCMC is to consider a random walk in the number of particles. In general, the probabilities for the creation of a new particle (at a specific position) or the destruction of an existing particle depend on the number of particles in the simulation volume and on their configuration. Following Im et al. [25] we here derive an expression for the creation or destruction of

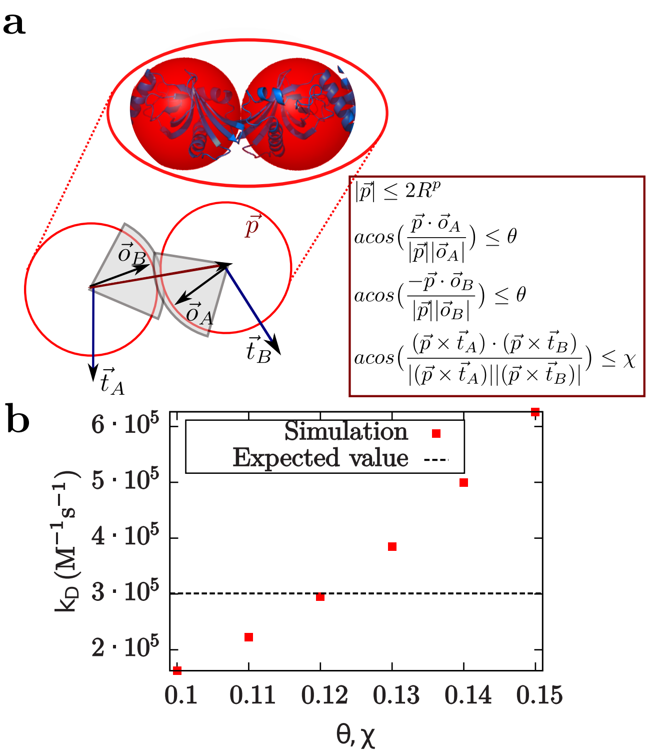

Figure S 3. (a) Model for the encounter between two single N-term domains. The encounter constraints are the same as for the full SAS-6 homodimer (Eq. S11-Eq. S14). (b) Diffusive on-rates based on Eq. S18 for a fixed patch radius $R^p = 2.05$ as a function of the angular constraints $\theta = \chi$. Here a minimum timestep of $\Delta t = 0.0001$ns has been used. The black line corresponds to the result predicted by the TransComp webserver for the diffusive on-rate of two N-term domains based on PDB structure 3Q0Y [28] at physiological salt conditions.

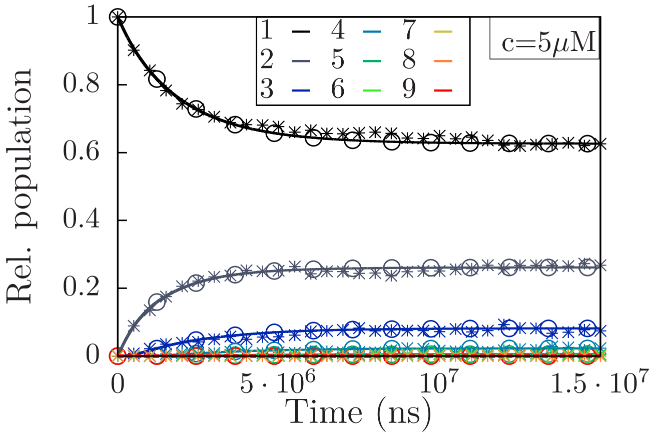

Figure S 4. Cluster population dynamics. Star symbols show averaged simulation data. Solid lines show the results using the rate equation approach [16] for the larger encounter volume (Tab. SIV and Tab. SV). Empty circles show the results from the rate equation approach using the smaller encounter volume (Tab. SII and Tab. SIII) when using a rescaled time $t_{\rm sc} = \alpha \times t \approx 123.3 \times t$.

new clusters which is adapted to our purposes. In the following we consider the chemical potential of an ideal gas $\mu_{\rm id} = -k_B T \ln\left(\frac{V}{\Lambda(T)^3 N}\right)$ (compare e.g. reference [27]) as the reference chemical potential, and $\Delta\mu$ denotes the difference from the ideal case. Here $\Lambda(T)$ is the thermal wavelength, V volume and N particle number. In the ideal case the particle concentration $n_{\rm id}$ is related to the chemical potential by $n_{\rm id} = N_{\rm id}/V = \Lambda(T)^{-3} e^{\beta\mu_{\rm id}}$ where β is inverse temperature $1/(k_B T)$. Given this relation the grand canonical partition sum can be written as [25, 27]:

$$\Xi(V,T,\mu) = \sum_{N=0}^{\infty} \frac{N_{\rm id}^N}{N!} \int d^{3N}x e^{-\beta W(\mathbf{x},N)} e^{\beta\Delta\mu N} \quad \text{with } x = \frac{r}{V^{1/3}}. \tag{S19}$$

The probability to find the system in state $(\mathbf{x}, N)$ is given by:

$$P(\mathbf{x},N) d^{3N}x = \frac{1}{\Xi} \times \frac{N_{\rm id}^N}{N!} e^{-\beta(W(\mathbf{x},N)-\Delta\mu N)} d^{3N}x. \tag{S20}$$

From Eq. S20 the ratio of the transition probabilities governing the creation or destruction of a new particle can be inferred by requiring detailed balance [25]. For typical concentrations used here the system can be considered as dilute. In this case Eq. S20 simplifies to the expected Poisson distribution describing the fluctuation of proteins in a finite observation volume.

$$P(N) \underbrace{\approx}_{\Delta W = \Delta\mu \approx 0} \frac{1}{\Xi} \frac{\langle N \rangle^N}{N!} = \frac{\langle N \rangle^N}{N!} e^{-\langle N \rangle}. \tag{S21}$$

In Eq. S21 the average number of proteins $\langle N \rangle = n_r V$, where n_r is the particle concentration of the repository and V is the size of the simulation volume. Requiring detailed balance we can infer the ratio of the transition probabilities for particle creation and destruction from Eq. S21:

$$P(N) p_{N\to N+1} = P(N+1) p_{N+1\to N} \tag{S22}$$

$$\Rightarrow \frac{p_{N\to N+1}}{p_{N+1\to N}} = \frac{\langle N \rangle}{N+1}. \tag{S23}$$

Choosing a normalization of $C = N + 1 + \langle N \rangle$ we obtain the following probabilities for the creation or destruction of a new particle:

$$p_{N\to N+1}^{\rm create} = \frac{\langle N \rangle}{N + 1 + \langle N \rangle} \tag{S24}$$

$$p_{N\to N-1}^{\rm destroy} = \frac{N}{N + \langle N \rangle}. \tag{S25}$$

As the scaffolding cylinder is localized in the x-y plane, we specify an inner region of interest (blue box in Fig. S2a) and restrict the unphysical GCMC moves to a volume $V_{\rm GCMC}$ far away from this region as has been previously argued [24–26]. In order not to bias

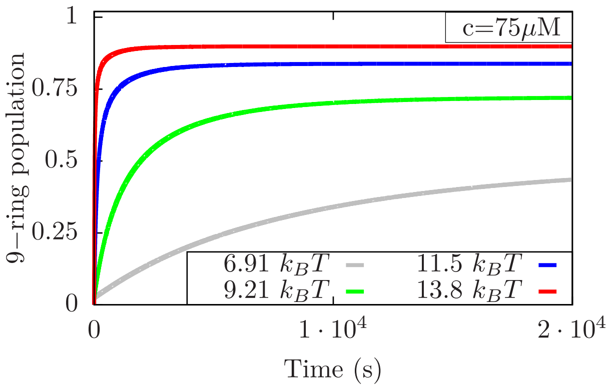

Figure S 5. The effect of different stabilization energies for the final ring structure for an initial homodimer concentration $c = 75\mu$M.

our BD simulations by the exchange dynamics with the reservoir we require that the exchange with the bulk is fast, compared to the time needed for a cluster to diffuse through $V_{\rm GCMC}$. Thus, the time between subsequent GCMC steps $t_{\rm GCMC}$ has to fulfill the requirement $t_{\rm GCMC} \ll L^2/D$ (compare Oberholzer et al. [24]), where D is the average translational diffusion coefficient of the cluster and L is the characteristic length scale associated with $V_{\rm GCMC}$. Based on the transition probabilities defined in Eq. S24 and Eq. S25, we can now couple our BD simulation volume to a bulk repository. To this end we use the following algorithm. In each GCMC move, either the creation or the destruction of a cluster is attempted with equal probability. The creation of a new cluster in $V_{\rm GCMC}$ is accepted with probability $p^{\rm create}_{N\to N+1}$ while the destruction of an existing cluster in $V_{\rm GCMC}$ is accepted with probability $p^{\rm destroy}_{N\to N-1}$. In this case the average number of proteins is given by: $\langle N\rangle = n_r V_{\rm GCMC}$. If the creation of a new cluster is accepted, a random position and orientation of the new cluster is chosen with the requirements that the center of the cluster is located inside $V_{\rm GCMC}$ and that no overlap with an existing cluster occurs. If an overlap is observed, a new position and orientation is chosen. Thus, in contrast to the work by Im et al. [25], where such an overlap would lead to a rejection of the complete GCMC step, we do not reject the GCMC move but instead choose a new position and orientation for the created protein. In this way the desired concentration of proteins in the simulation volume is established even when a non-vanishing fraction (dilute limit) of the simulation volume is occupied by other clusters.

In Fig. S2a, the 2D projection of a simulation volume of size $V_{\rm sim} = (100{\rm nm})^3$ is shown, which is coupled to a repository by GCMC steps. For simplicity we here consider only non-reactive, spherical proteins. A generalization to interacting proteins will be discussed later. In the setup shown in Fig. S2a, the GCMC steps are restricted to a region of width $L = 5$nm from the x-y-boundary, and gray marks depict the center of the newly created proteins. In addition, the inner region containing the scaffold, which is localized in the x-y direction, is marked in blue. This inner region has a volume of $V_{\rm inner} = 1/5 \times V_{\rm sim}$ with the z-extension of the inner region spanning the full box height. In Fig. S2b and Fig. S2c, the number of particles observed in the inner region (blue) is compared to the expected Poisson distribution (red) for two different particle concentrations. We see that the number of proteins observed in our simulations agrees well with the expected distribution, albeit in the simulations the distribution seems to be shifted to slightly larger values (e.g in Fig. S2c $\langle N\rangle_{\rm sim} = 10.046$ instead of the expected 10.0). Despite these very small deviations, the coupling mechanism presented here is well suited to study the effect of a localized structure like a scaffold which is embedded in a large system without the need to simulate the complete system.

For the coupling to a bulk repository of SAS-6 homodimers one further has to take into account that, depending on the homodimer concentration of the repository, a certain fraction of larger oligomers exists in the bulk and we calculate the equilibrium concentration $n_{r,k}$ of clusters of size k for a given total homodimer concentration n_r using the previously defined rate equation approach (ref. [16]). Thus, in order to take the existence of higher oligomers in the bulk into account in our GCMC scheme, we consider each oligomer as an individual species. During each GCMC step the creation or the destruction of each of the oligomer species ($k = 1, \cdots, 9$) is attempted according to Eq. S24 or Eq. S25, respectively, with $< N_k >^{\rm GCMC} = n_{r,k} \times V_{\rm GCMC}$ being the expected number of oligomers of size k in the GCMC region $V_{\rm GCMC}$.

IV. CHOICE OF PATCH PARAMETERS

Due to the lack of experimental knowledge of the speed of the reaction process, we instead rely on the TransComp webserver provided by the Zhou group [29–31] to estimate the reaction speed. This webserver is set up to predict the reaction rate constant from the crystal structure of a given complex. In order to estimate the reaction rate constant for the dimerization of individual N-term complexes, we use part of the crystal structure with PDB entry 3Q0Y [28] corresponding to one N-term complex as input for the TransComp webserver. Under physiological salt conditions a diffusive reaction rate constant of $k_D^{N,N} \approx 3 \times 10^5 {\rm M}^{-1}{\rm s}^{-1}$ has been predicted by the webserver for the two N-term domains with a mild influence of electrostatics only. However, exchanging the order in which the chains of the N-term complex are provided to the webserver results in rather large changes in the estimated rate constants (by about a factor of 4). Thus, the value predicted for the reaction rate constant of the dimerization of two N-terms can only be considered as a rough estimate which sets the overall time scale of the assembly process. Nevertheless, here we choose

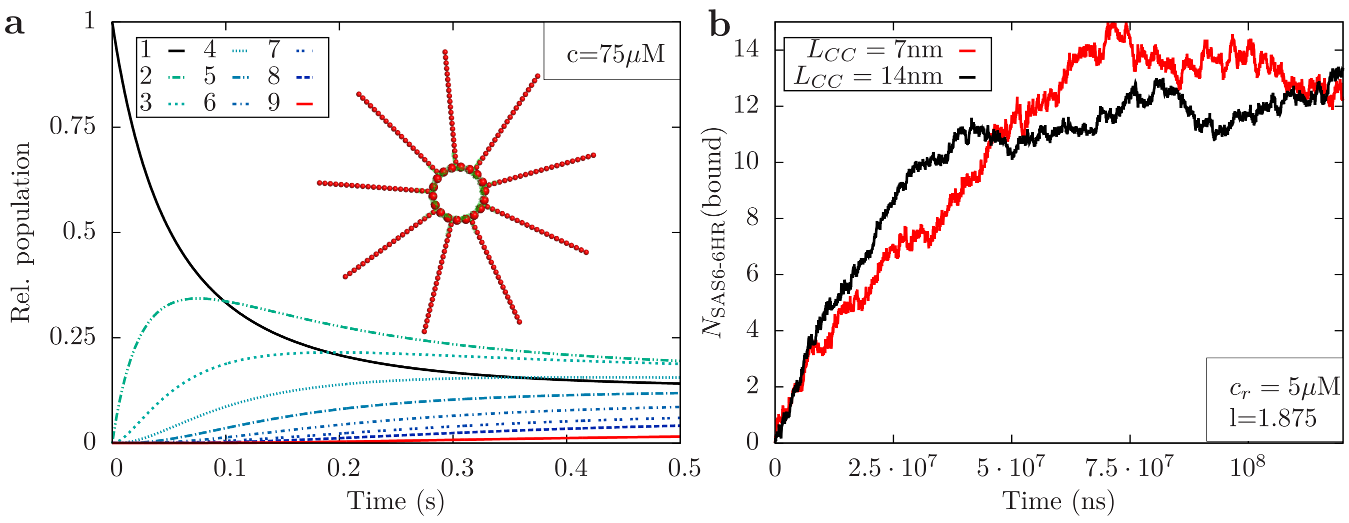

Figure S 6. Effect of longer CC domain. (a) Bulk-assembly dynamics for a SAS-6 model with the CC-domain being elongated to $\approx$ 50nm for a homodimer concentration of $c = 75\mu$M (no internal stabilization, parameter set 1). (b) Number of SAS-6 homodimers targeted to a cylindrical scaffold (parameter set 2 with scaffold as described in the main text) for an interaction strength of $l = 1.875$ and a reservoir concentration of $c_r = 5\mu$M. The red line corresponds to the SAS-6-6HR model with a CC length $L_{CC} \approx$ 7nm while the black line shows the number of SAS-6 homodimer targeted to the cylinder when elongating the CC domain to $L_{CC} \approx$ 14nm.

our patch parameters accordingly to this estimate and investigate the effect of oligomer size for the reaction rate constants. We describe an individual N-term domain by a single hard sphere equipped with a reactive patch whose center coincides with the center of the N-term domain (see Fig. S3a). The constraints defining an encounter between two N-term domains are identical to those used for the full SAS-6 model including the torsional constraint (Eq. S14). Assuming only short ranged interactions (surface reactivity in the spirit of reactive boundaries), we choose a patch radius of $R^p = 2.05$nm, which is only slightly larger than the radius of the hard sphere modeling the N-term ($R_N = 1.979$nm).

Additionally we require that $\theta = \chi$ in order to eliminate one further parameter. With this choice we now vary θ (and simultaneously χ) and determine the diffusive reaction rate constant between two spherical N-terms according to the algorithm described by Zhou and coworker [19, 20]. The result is shown in Fig. S3b. Choosing $\theta = \chi = 0.12$ rad, we find good agreement between the expected diffusive rate constant and the one observed with our patchy sphere model. With this choice of patch parameters, the encounter volume for the dimerization reaction between two N-terms is $V^{\text{NN}^\star} = V^{\text{SAS-6}^\star}_{A_i,B_j} \approx 1.43 \times 10^{-5}\text{nm}^3$ (see Eq. S15), and the encounter volume for the reaction of two complete SAS-6 homodimers is $V^{\text{SAS-6}^\star}_{1,1} \approx 5.7 \times 10^{-5}\text{nm}^3$ (see Eq. S16). Here it has to be noted that this choice of patch parameters correctly reproduces the value estimated by the TransComp webserver, although it is not unique.

Considering that the estimated reaction rate constant for the dimerization of two N-term complexes is about four to five orders of magnitude below the Smoluchowski rate constant for two spheres of similar size and that a very small timestep is required to appropriately resolve the small encounter volume, direct simulations of the assembly process are not feasible. Thus, in order to investigate bulk assembly taking the effect of slower diffusion of larger oligomers into account, we instead rely on the previously introduced rate equation approach for the assembly of ring structures [16]. To use this rate equation approach, the size of the encounter volumes as well as the diffusive reaction rate constants for all combinations of ring fragments containing i and j homodimers need to be determined. While for the reaction of clusters of size $i + j < 9$ the encounter volume is the same as for the reaction of two homodimers, we have to take into account that for the reaction of clusters with size $i + j = 9$ the encounter volume is reduced due to steric collisions and double encounters between the clusters. The MC scheme described for this purpose in reference [16] cannot be applied here to determine the reduction of the accessible encounter volume for the formation of complete rings due to the large difference between the box volume necessary to prevent artifacts ($V_{\text{box}} \gtrsim (40\text{nm})^3 \approx 6.4 \times 10^4\text{nm}^3$) and the expected encounter volume size $V^{\text{SAS-6}^\star}_{i,j} \leq 5.7 \times 10^{-5}\text{nm}^3$. In this case more than 10^9 trials are needed until one encounter is sampled. Here we circumvent this problem using a slightly different MC scheme. In particular, we sample only encounter configurations between two ring fragments (fulfilling the constraints given in Eq. S11- Eq. S14), but we neglect any steric interactions. Sampling N encounter configurations we record the fraction of accessible configurations with-

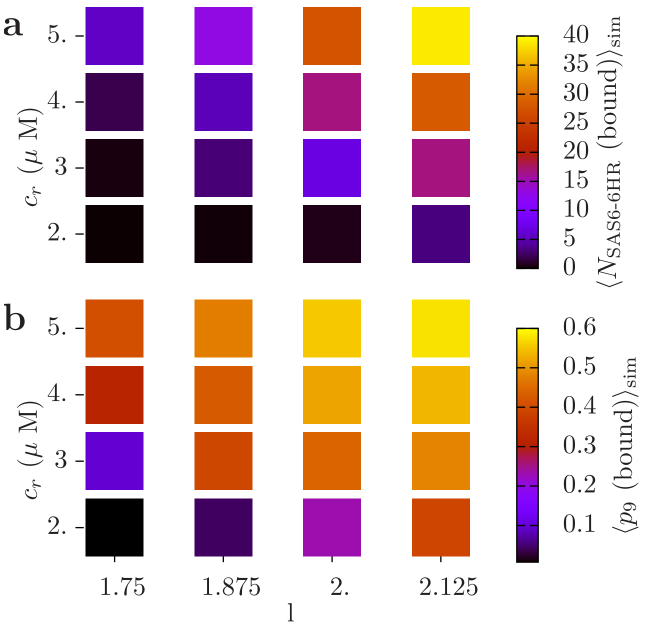

Figure S 7. Number of SAS-6-6HR homodimers (a) and relative population p_9 of complete SAS-6-6HR rings (b) observed in steady state as a function of the interaction strength l for different repository concentrations c_r.

out a steric overlap $f^{i,j}_{\mathrm{access}} = (N - N^{i,j}_{\mathrm{steric}})/N$ as well as the fraction of the accessible configurations for which a simultaneous overlap of two pairs of patches occurs $f^{i,j}_{\mathrm{double}} = N^{i,j}_{\mathrm{double}}/(N - N^{i,j}_{\mathrm{steric}})$. As we know the size of the encounter volume for the reaction of two homodimers (compare Eq. S16), we can infer the reduction of the encounter volume for larger ring fragments combining into a complete ring due to steric blocking and overlapping encounter volumes of different patch combinations by:

$$V^{\mathrm{SAS6}^\star}_{i,j} = V^{\mathrm{SAS\text{-}6}^\star}_{1,1}\,\frac{f^{i,j}_{\mathrm{access}} - 0.5 f^{i,j}_{\mathrm{access}} f^{i,j}_{\mathrm{double}}}{f^{1,1}_{\mathrm{access}}}. \qquad \text{(S26)}$$

The factor of 1/2 reflects the fact that simultaneous double encounters between two clusters are not allowed in our simulations and only one of the encounters is considered. In the case studied here, $f^{i,j}_{\mathrm{double}} \ll 1$ so that the double-encounter configurations lead only to a minor reduction of $V^{\mathrm{SAS\text{-}6}^\star}_{i,j}$ compared to the effect of steric exclusion. The resulting encounter volumes for the reaction between different SAS-6 ring fragments are given in Tab. SII. Finally, after having estimated the encounter volumes for the reaction between different ring fragments we compute the diffusive reaction rate constant from many short BD trajectories with a minimum timestep of $\Delta t = 0.0001$ns each starting with a random encounter configuration of the two cluster fragments. The diffusive on-rates follow from Eq. S18 and their dependence on the oligomerization state is given in Tab. SIII.

While using the patch parameters estimated by matching the TransComp predictions allows to run short simulations from which diffusive on-rates used in our rate equation approach can be inferred, direct simulations of the whole assembly process are prohibited by the small size of the encounter volume and the related slow assembly kinetics. Thus, in order to be able to study the effect of a localized scaffold for the assembly of SAS-6 rings, we simulate the assembly with enhanced diffusive reaction dynamics by relaxing the constraints defining an encounter. In particular, we use a patch radius of $R^p_{\mathrm{enh}} = 2.15$nm and orientational constraints of $\theta_{\mathrm{enh}} = \chi_{\mathrm{enh}} = 0.4$rad. This results in larger encounter volumes between different ring fragments, which are given in Tab. SIV. The enlarged encounter volumes reduce the time needed to establish an encounter. This is reflected by the diffusive on-rate constants calculated with the relaxed encounter constraints. The diffusive association rate constants are shown in Tab. SV. Comparing the relative change in the diffusive on-rate constants for different cluster sizes ($k^{i,j}_D/k^{1,1}_D$) calculated for the SAS-6-6HR model with the smaller encounter volumes (Tab. SIII) and the enlarged encounter volumes (Tab. SV), we see that they are very similar except for the final step leading to ring closure ($i + j = 9$). While the final ring formation step is crucially affected by steric blocking, the relative reduction of the diffusive on-rate constants between two smaller fragments ($i + j < 9$) is almost independent of the increased encounter volume and depends only on the size and shape of the two clusters. Thus, increasing the encounter volume can be considered as rescaling of time by a factor $\alpha \approx 123.3$ which is defined by the ratio of $k^{1,1}_D$ estimated for the larger and the smaller encounter volume. This can be seen in Fig. S4 where the evolution of the relative cluster population for the assembly of SAS-6-6HR homodimers is shown starting with an initial concentration of $c = 5\mu$M. Here, solid lines show the resulting cluster populations observed with our rate equation approach using the larger encounter volumes (Tab. SIV) and the corresponding rate constants (Tab. SV). Empty circles show the resulting cluster populations using the smaller encounter volumes (Tab. SII) and the corresponding rate constants (Tab. SIII) when rescaling time ($t_{\mathrm{sc}} = \alpha t$). Additionally the evolution of the relative cluster population observed in direct simulations ($\Delta t = 0.01$ns, 30 independent trajectories) starting with 125 SAS-6-6HR homodimers in a periodic box of size $V = 346\mathrm{nm}^3$ is shown by star symbols. For the simulations and the rate equation approach with the larger encounter parameters a microscopic reaction rate of $k_a = 4\mathrm{ns}^{-1}$ has been used, resulting in strongly diffusion-influenced assembly, and the microscopic dissociation rate constant follows from Eq. S17. In the case of the smaller encounter volumes, the microscopic rates are chosen appropriately so that the assembly is equally diffusion-influenced (the ratio $k_a/k^{1,1}_{D,b}$ stays preserved where $k^{1,1}_{D,b}$ is the diffusive back reaction rate constant for two single homodimers). From Fig. S4 one can clearly see that in the case of bulk assem-

bly enlarging the encounter volume can be considered as rescaling of time. Moreover, direct simulations of the assembly process are feasible when relaxing the encounter constraints. As expected, the evolution of the relative cluster populations observed by direct simulations shows excellent agreement with the relative cluster distributions predicted from our rate equation approach.

V. SUPPLEMENTARY MOVIES

Movie 1 shows bulk assembly at homodimer concentration $c = 75\mu$M. One clearly sees the variability in the intermediates structures assembled. Movie 2 shows assembly around a freely accessible spherical scaffold at $c = 5\mu$M. One sees many intermediates that obstruct each other. Movie 3 shows assembly around an embedded spherical scaffold at $c = 5\mu$M. One sees how a complete ring is readily formed.

VI. TABLES

Parameters	Set 1 (normal)	Set 2 (accelerated)
R^p (nm)	2.05	2.15
θ ,χ (rad)	0.12	0.4
k_a (ns^{-1})	10^4	4
k_d (ns^{-1})	2.6×10^{-6}	1.056×10^{-6}
Δt (ns)	0.0001	0.01

Table S I. Simulation parameters. Sets 1 and 2 were used to simulate bulk assembly and scaffold-assisted assembly, respectively.

Size	1	2	3	4	5	6	7	8
1	1	1	1	1	1	1	1	0.55
2	1	1	1	1	1	1	0.59	-
3	1	1	1	1	1	0.59	-	-
4	1	1	1	1	0.62	-	-	-
5	1	1	1	0.62	-	-	-	-
6	1	1	0.59	-	-	-	-	-
7	1	0.59	-	-	-	-	-	-
8	0.55	-	-	-	-	-	-	-

Table S II. Weight matrix M^V for different SAS-6 ring fragments with patch parameters $R^p = 2.05$nm, $\theta = \chi = 0.12$rad. The encounter volume for a specific fragment combination is given by $V^\star_{i,j} = V^\star_{1,1} M^V_{i,j}$ with $V^\star_{1,1} = 5.8 \times 10^{-5}$nm^3 being the encounter volume for two SAS-6 homodimers calculated according to Eq. S15 and Eq. S16. The volume reduction for ring fragments combining into a full ring has been estimated based on Eq. S26.

Size	1	2	3	4	5	6	7	8
1	1	0.83	0.75	0.71	0.68	0.65	0.62	0.27
2	0.83	0.66	0.59	0.54	0.51	0.48	0.26	-
3	0.75	0.59	0.53	0.48	0.44	0.25	-	-
4	0.71	0.54	0.48	0.43	0.25	-	-	-
5	0.68	0.51	0.44	0.25	-	-	-	-
6	0.65	0.48	0.25	-	-	-	-	-
7	0.62	0.26	-	-	-	-	-	-
8	0.27	-	-	-	-	-	-	-

Table S III. Weight matrix M^{k_D} estimated based on the algorithm by Zhou[19, 20] for different ring fragment combinations of SAS-6-6HR homodimers (shorter CC) with patch parameters $R^p = 2.05$nm, $\theta = \chi = 0.12$rad. The diffusive on-rates for the different ring fragments are given by $k_D^{i,j} = k_D^{1,1} M^{k_D}_{i,j}$ with $k_D^{1,1} = 8.3 \times 10^{-4}$nm^{3}ns^{-1} being the diffusive on-rate for two SAS-6-6HR homodimers.

Size	1	2	3	4	5	6	7	8
1	1	1	1	1	1	1	1	0.79
2	1	1	1	1	1	1	0.80	-
3	1	1	1	1	1	0.81	-	-
4	1	1	1	1	0.79	-	-	-
5	1	1	1	0.79	-	-	-	-
6	1	1	0.81	-	-	-	-	-
7	1	0.80	-	-	-	-	-	-
8	0.79	-	-	-	-	-	-	-

Table S IV. Weight matrix M^V for different SAS-6 ring fragments with enhanced reactivity ($R^p = 2.15$nm, $\theta = \chi = 0.4$rad). The encounter volume for a specific fragment combination is given by $V^\star_{i,j} = V^\star_{1,1} M^V_{i,j}$ with $V^\star_{1,1} = 0.0585$nm^3 being the encounter volume for two SAS-6 homodimers calculated according to Eq. S15 and Eq. S16. The volume reduction for ring fragments combining into a full ring has been estimated based on Eq. S26.

Size	1	2	3	4	5	6	7	8
1	1	0.82	0.76	0.72	0.68	0.65	0.62	0.42
2	0.82	0.67	0.61	0.56	0.52	0.49	0.36	-
3	0.76	0.61	0.52	0.48	0.42	0.33	-	-
4	0.72	0.56	0.48	0.42	0.31	-	-	-
5	0.68	0.52	0.42	0.31	-	-	-	-
6	0.65	0.49	0.33	-	-	-	-	-
7	0.62	0.36	-	-	-	-	-	-
8	0.42	-	-	-	-	-	-	-

Table S V. Weight matrix M^{k_D} estimated based on the algorithm by Zhou[19, 20] for different ring fragment combinations of SAS-6-6HR homodimers (shorter CC) with enhanced reactivity ($R^p = 2.15$nm, $\theta = \chi = 0.4$rad). The diffusive on-rates for the different ring fragments are given by $k_D^{i,j} = k_D^{1,1} M^{k_D}_{i,j}$ with $k_D^{1,1} = 0.102$nm^{3}ns^{-1} being the diffusive on-rate for two SAS-6-6HR homodimers.

[1] C. B. Korn and U. S. Schwarz. *Mean first passage times for bond formation for a Brownian particle in linear shear flow above a wall.* The Journal of Chemical Physics, 126(9):095103 (2007).

[2] J. Schluttig, D. Alamanova, V. Helms and U. S. Schwarz. *Dynamics of protein-protein encounter: A Langevin equation approach with reaction patches.* The Journal of Chemical Physics, 129:155106 (2008).

[3] J. Schluttig. *Stochastic dynamics of protein assembly.* Ph.D. thesis, Ruprecht-Karls-Universität Heidelberg (2009).

[4] J. G. de la Torre and V. A. Bloomfield. *Hydrodynamic properties of macromolecular complexes. I. Translation.* Biopolymers, 16(8):1747 (1977).

[5] B. Carrasco and J. G. de la Torre. *Improved hydrodynamic interaction in macromolecular bead models.* The Journal of Chemical Physics, 111(10):4817 (1999).

[6] D. L. Ermak and J. A. McCammon. *Brownian dynamics with hydrodynamic interactions.* The Journal of Chemical Physics, 69(4):1352 (1978).

[7] F. C. Collins and G. E. Kimball. *Diffusion-Controlled Reaction Rates.* Journal of Colloid Science, 4(4):425 (1949).

[8] M. Eigen. *Diffusion control in biochemical reactions. In Quantum Statistcal Mechanics in the Natural Sciences*, vol. 4 of *Studies in the Natural Sciences.* Plenum Press, New York and London (1974).

[9] D. Shoup and A. Szabo. *Role of diffusion in ligand binding to macromolecules and cell-bound receptors.* Biophysical Journal, 40(1):33 (1982).

[10] S. H. Northrup and H. P. Erickson. *Kinetics of protein-protein association explained by Brownian dynamics computer simulation.* Proceedings of the National Academy of Sciences, 89(8):3338 (1992).

[11] N. Agmon and A. Szabo. *Theory of reversible diffusion-influenced reactions.* The Journal of Chemical Physics, 92:5270 (1990).

[12] R. R. Gabdoulline and R. C. Wade. *Simulation of the Diffusional Association of Barnase and Barstar.* Biophysical Journal, 72(5):1917 (1997).

[13] R. Alsallaq and H.-X. Zhou. *Prediction of Protein-Protein Association Rates from a Transition-State Theory.* Structure, 15(2):215 (2007).

[14] G. Schreiber, G. Haran and H.-X. Zhou. *Fundamental Aspects of Protein-Protein Association Kinetics.* Chemical Reviews, 109(3):839 (2009).

[15] D. Shoup and A. Szabo. *Role of Diffusion in Ligand Binding to Macromolecules and Cell-Bound Receptors.* Biophysical Journal, 40(1):33 (1982).

[16] H. C. R. Klein and U. S. Schwarz. *Studying protein assembly with reversible Brownian dynamics of patchy particles.* The Journal of Chemical Physics, 140(18):184112 (2014).

[17] S. S. Andrews and D. Bray. *Stochastic simulation of chemical reactions with spatial resolution and single molecule detail.* Physical Biology, 1(3):137 (2004).

[18] D. T. Gillespie. *Exact Stochastic Simulation of Coupled Chemical Reactions.* The Journal of Physical Chemistry, 81(25):2340 (1977).

[19] H.-X. Zhou. *Kinetics of Diffusion-Influenced Reactions Studied by Brownian Dynamics.* Journal of Physical Chemistry, 94(25):8794 (1990).

[20] H.-X. Zhou and A. Szabo. *Theory and Simulation of the Time-Dependent Rate Coefficients of Diffusion-Influenced Reactions.* Biophysical Journal, 71(5):2440 (1996).

[21] T. Geyer, C. Gorba and V. Helms. *Interfacing Brownian dynamics simulations.* The Journal of Chemical Physics, 120(10):4573 (2004).

[22] P. Bauler, G. A. Huber and J. A. McCammon. *Hybrid finite element and Brownian dynamics method for diffusion-controlled reactions.* The Journal of Chemical Physics, 136(16):164107 (2012).

[23] B. Franz, M. B. Flegg, S. J. Chapman and R. Erban. *Multiscale Reaction-Diffusion Algorithms: PDE-Assisted Brownian Dynamics.* SIAM Journal on Applied Mathematics, 73(3):1224 (2013).

[24] M. R. Oberholzer, N. J. Wagner and A. M. Lenhoff. *Grand canonical Brownian dynamics simulation of colloidal adsorption.* The Journal of Chemical Physics, 107(21):9157 (1997).

[25] W. Im, S. Seefeld and B. Roux. *A Grand Canonical Monte Carlo–Brownian Dynamics Algorithm for Simulating Ion Channels.* Biophysical Journal, 79(2):788 (2000).

[26] B. Roux, T. Allen, S. Berneche and W. Im. *Theoretical and computational models of biological ion channels.* Quarterly Reviews of Biophysics, 37(01):15 (2004).

[27] D. A. McQuarrie. *Statistical Mechanics.* Harper's chemistry series. Harper & Row, New York [u.a.] (1976).

[28] D. Kitagawa, I. Vakonakis, N. Olieric, M. Hilbert, D. Keller, V. Olieric, M. Bortfeld, M. C. Erat, I. Flückiger, P. Gönczy and M. O. Steinmetz. *Structural Basis of the 9-Fold Symmetry of Centrioles.* Cell, 144(3):364 (2011).

[29] R. Alsallaq and H.-X. Zhou. *Electrostatic rate enhancement and transient complex of protein–protein association.* Proteins: Structure, Function, and Bioinformatics, 71(1):320 (2008).

[30] S. Qin and H.-X. Zhou. *Prediction of Salt and Mutational Effects on the Association Rate of U1A Protein and U1 Small Nuclear RNA Stem/Loop II.* The Journal of Physical Chemistry B, 112(19):5955 (2008).

[31] S. Qin, X. Pang and H.-X. Zhou. *Automated Prediction of Protein Association Rate Constants.* Structure, 19(12):1744 (2011).
